# Comparative Study on Two Pretreatment Processes for Chemical Phase Analysis of Gold in Geological Samples by Atomic Absorption Spectrometry

**DOI:** 10.1155/2019/1792792

**Published:** 2019-07-10

**Authors:** Xiaodan Tang, Hang Li, Hongyan Liu, Bing Li, Yuyan Zhao, Jilong Lu, Jian Zhou, Qingqing Liu

**Affiliations:** ^1^Key Laboratory of Geochemical Exploration, Ministry of Land and Resources, Institute of Geophysical and Geochemical Exploration, Chinese Academy of Geological Sciences, Langfang 065000, China; ^2^College of Geo-exploration Science and Technology, Jilin University, Changchun 130026, China; ^3^UNESCO International Centre on Global-Scale Geochemistry, Langfang 065000, China

## Abstract

Sample pretreatment is important for chemical phase analysis of elements. In this study, the geological samples of the Laozuoshan gold mine are chosen to pretreat by ultrasonic centrifugation and cyclotron oscillation, and the content of gold in eight chemical phases (water-soluble, ion exchange and clay adsorption, organic matter bound, iron-manganese oxide bound, naked or seminaked, carbonate bound, sulfide bound, and insoluble silicate states) is determined by atomic absorption spectrometry. The results show that the gold content of water-soluble, ion exchange and clay adsorption, iron-manganese oxide, and naked or seminaked states in the rock and ore samples is low, and some samples have high gold content of insoluble silicate states in the two methods. However, the gold content of organic matter bound, carbonate bound, and sulfide bound states obtained by ultrasonic centrifugation and cyclotron oscillation methods is significantly different. According to the X-ray fluorescence spectrometry data and the actual geological condition, the result given by the cyclotron oscillation method is more reasonable. The gold content of sulfide bound state in sediment samples is the highest and consistent with the mineral information, which could be applied to preliminarily predict the rock and ore conditions in the corresponding mining areas. In contrast with ultrasonic centrifugation, the cyclotron oscillation method has the advantages of simplicity, high efficiency, practicality, and environmental protection, and it can be better used for the determination of gold chemical phase state in geological samples by atomic absorption spectrometry.

## 1. Introduction

Gold is an extraordinary mineral resource with important status and wide application in geology [1, 2], electronics [3, 4], chemical [5, 6], and medical [7, 8] fields. However, it has low content and uneven distribution in the earth's crust, which makes it difficult to analyze. In recent years, gold mineral resources are increasingly depleted under continuous development and utilization. Searching for hidden gold mines and re-extraction of gold in tailings has become a hot spot for exploration and research. For the rational, effective, and comprehensive development and utilization of gold mineral resources, it is necessary to explore the occurrence status of gold in various geological samples.

Chemical phase analysis [9], one of the important means to ascertain the occurrence states of elements, enables the separation and determination of different phase elements by selective solvent dissolution or leaching of one or a class of minerals. The predecessors have done some research on the chemical phase analysis of gold. The phase of gold in geochemical samples was initially divided into six, namely, natural gold, carbonate phase, pyrite wrapped phase, oxide phase, sulfide phase, and quartz-silicate combination phase [10]. In consideration of differences in landscape geochemical conditions and biogeochemical effects, the chemical phase of gold was increased to eight by introducing clay absorbed phase (exchangeable phase) and organic phase [11]. Then, the existence forms of “invisible gold” in several complex ores and the analytical methods for the determination of gold in different chemical phases were summarized [12, 13]. The selective solvents required for chemical phase analysis of gold ore were explored in experiments [14]. Furthermore, gold chemical phase analysis is applied to the geological analysis of different mines to explore its indication significance for prospecting [15, 16].

In order to accurately, quickly, and effectively determine the content of phase gold in geological samples, the continuous improvement and innovation of analytical techniques are particularly important. In the phase analysis process, sample pretreatment [17–20] is one of the key steps, which seriously affects the accuracy of the results and directly determines the success of the experiment or not. At the same time, the test methods will also have a certain impact on the experimental results. Commonly used gold test methods include titration [21], atomic absorption spectrometry (AAS) [20, 22–24], inductively coupled plasma optical emission spectroscopy [25, 26], and inductively coupled plasma mass spectrometry [27, 28]. Among them, the AAS meets the test conditions of most geological samples, and its analytical techniques are relatively mature and widely used [29–32]. Therefore, the AAS was used here to determine the gold content.

The main purpose of this study was to explore, contrast, and validate the pretreatment process applicable to the gold chemical phase analysis in geological samples by AAS. Firstly, the geological samples of the Laozuoshan gold deposit were chosen to analyze the gold-related elements by X-ray fluorescence spectrometry (XRF) [33, 34] to preliminarily predict the content and occurrence state of gold. Then, the samples were pretreated by ultrasonic centrifugation and cyclotron oscillation methods for chemical phase analysis, and the content of gold in eight phases (water-soluble, ion exchange and clay adsorption, organic matter bound, iron-manganese oxide bound, naked or seminaked, carbonate bound, sulfide bound, and insoluble silicate states) was determined by AAS, which filled the gap in the chemical phase analysis of the Laozuoshan gold mine. Finally, the two pretreatment processes were compared and evaluated by combining the XRF data and geological and geochemical characteristics of the Laozuoshan gold deposit [35, 36].

## 2. Materials and Methods

### 2.1. Reagents and Standards

Hydrochloric acid (HCl), nitric acid (HNO_3_), and hydrofluoric acid (HF) in guaranteed reagent grade and ultrapure deionized water with a resistivity of 18.2 MΩ·cm at 25°C were used for sample preparation. The other reagents were of analytical grade. The main reagents prepared in the experiment were 50 g/L ammonium citrate solution, 4 g/L sodium hydroxide (NaOH)-40 g/L sodium pyrophosphate (Na_4_P_2_O_7_) mixed solution, 100 g/L ammonium citrate-40 g/L hydroxylamine hydrochloride mixed solution (pH ≈ 7 with ammonia), 5 g/L iodine (I_2_)-15 g/L potassium iodide (KI) mixed solution (pH ≈ 10 with ammonia), 4 mol/L acetic acid (HAc) solution, 5 mL/L bromine (Br_2_)-100 g/L sodium chloride (NaCl) mixed solution, 250 g/L ferric chloride (FeCl_3_) solution (with 1% HCl), 10 g/L thiourea solution (with 1% HCl), and aqua regia.

National standard gold single element solution (GSB 04-1715-2004, 1000 *μ*g/mL) in 1.5 mol/L HCl was obtained from National Center of Analysis and Testing for Nonferrous Metals and Electronic Materials. The gold standard solutions required in the experiment were prepared by stepwise dilution. Four certified reference materials (CRMs) (GBW07247a, GBW07298a, GBW07105, and GBW07309) were acquired from the Institute of Geophysical and Geochemical Exploration, Chinese Academy of Geological Sciences (Langfang, China).

### 2.2. Instrumentation

Samples were triturated by a planetary ball mill (QM-3SP4, Laibu, China), weighed with an electronic balance (ATY124, Shimadzu, Japan), and ashed in a muffle furnace (SXL-1008, Jinghong, China). The electric heating plate (SB-1.8-4, Shanghai Shiyan, China) was applied in total gold digestion process. The cyclotron oscillator (HY-8A, Jintan Jingda, China), centrifuge (TDL-5A, Anting, China), ultrasonic cleaner (KQ-400KDE, Kunshan, China), and vacuum pump (SHB-III, Great Wall, China) devices were used for phase gold pretreatment. The constant-temperature water bath (HH-S26S, Jintan Instruments, China) was applied to the gold desorption. Some gold-related elements were determined by an X-ray fluorescence spectrometer (EDX 6000B, Tianrui, China). Gold determination was performed on an AAS (A3, Persee, China) instrument. The optimum operating parameters of the XRF, flame, and graphite furnace AAS are summarized in Table 1, and the graphite furnace heating process is shown in Table 2.

### 2.3. Sample Processing

The collected ore and sediment samples of the Laozuoshan gold deposit were dried, smashed by a crusher, finely grinded to powder by a planetary ball mill, filtered through a 200-mesh sieve, and placed in a desiccator for use.

### 2.4. Experimental Procedure of Total Gold

With reference to the geological and mineral industry standard DZ/T0279.19-2016 of the People's Republic of China, combined with the actual situation of the laboratory, the leaching experiment of total gold in geological samples was designed as the following two steps:

1. High-temperature ashing-acid digestion: Ten grams of sample were accurately weighed into a porcelain crucible, heated to 700°C in a muffle furnace, and held for 1.5 hours. After cooling, the sample was transferred to a Teflon Erlenmeyer flask (TEF), into which 50 mL 50% aqua regia and 10 mL HF were added. The TEF was placed on a hot plate and heated to dissolve the sample, and the solution was kept slightly boiled, evaporated to half, and then cooled.
2. Gold enrichment and desorption: 80 mL ultrapure deionized water, 3 mL FeCl_3_ solution, and a gold-absorbing foam (3 × 2 × 1 cm^3^, ∼0.35 g) were added to the resulting solution of the TEF, which was then placed on a cyclotron and shaken for 30 minutes at a frequency of 200 rpm. Subsequently, the foam was taken out from the flask, the residual acid and residue were rinsed with ultrapure deionized water, the water was drained with the filter paper, and the solution was placed in a 50 mL colorimetric tube in which 25 mL thiourea solution had been added. After 45 minutes in the boiling water bath, the foam was repeatedly pressed and taken out. The resulting solution was cooled to room temperature and to be measured.

At the same time, the same method was used for the blank control experiment.

### 2.5. Experimental Procedure of Phase Gold

Methods I and II represent ultrasonic centrifugation and cyclotron oscillation processes, respectively. Method I uses ultrasonic waves to propagate in the form of longitudinal waves within the liquid, cause the liquid molecules to vibrate at their equilibrium positions, and then increase the solubility of the sample in the solvent. When the ultrasonic power is strong enough, the cavitation even instantaneous high temperature and high pressure will generate. This extreme environment is easy to accelerate the internal motion or depolymerization of molecules and cause chemical reaction. Here, the samples were sonicated at the frequency of 40 kHz for 1 hour in an ultrasonic cleaner with the power of 200 W and the temperature of 25°C–30°C, and then centrifuged at 4500 rpm for 15 minutes in a centrifuge. Method II, also called the circumferential oscillation, is a 360° rotation oscillation on the horizontal surface. During the oscillation process, the oscillated liquid will appear in a swirl shape in the container and its turbulence is more intense, which allows the sample to be distributed more evenly throughout the extractant and thereby accelerates the diffusion rate of the extracted gold into the solution. Here the samples were shaken at the frequency of 200 rpm for 1.5 hours on a cyclotron oscillator with the power of 100 W and the amplitude of 20 mm.

#### 2.5.1. Water-Soluble State (WSS)

Method I: Ten grams of sample and 50 mL ultrapure deionized water were accurately taken into a centrifuge cup, shaken well, and sonicated in an ultrasonic cleaner for 1 hour. The temperature of the ultrasonic cleaner should be maintained at 25°C–30°C during the ultrasound process. Then the sample was centrifuged at 4500 rpm for 15 minutes and filtered through a 0.45 *μ*m membrane. The residue and filter flask were rinsed several times with ultrapure deionized water. The residue was retained in the original centrifuge cup for the gold extraction of the next phase. The filtrate was completely transferred to a TEF, heated, and concentrated to about 20 mL on a hot plate. 25 mL of aqua regia was added to the resulting filtrate in the TEF, shaken well, and heated to dissolve gold. The resulting solution was evaporated to about 20 mL and cooled. The subsequent operation was the same as Gold enrichment and desorption described in Section 2.4.

Method II: Ten grams of sample and 50 mL ultrapure deionized water were accurately taken into a conical flask with cover, shaken at 200 rpm for 1.5 hours on a cyclotron oscillator, and filtered through a 0.45 *μ*m membrane. The residue, conical flask, and filter flask were rinsed several times with ultrapure deionized water. The residue was retained in the original conical flask for the gold extraction of the next phase. The filtrate was completely transferred to a new TEF, and the subsequent operation was the same as Method I above.

#### 2.5.2. Ion Exchange and Clay Adsorption State (IECAS)

Fifty millilitres of 50 g/L ammonium citrate was added to the remained residue after the gold extraction of WSS, and the subsequent operations were the same as methods I and II of Section 2.5.1, respectively.

#### 2.5.3. Organic Matter Bound State (OMBS)

Fifty millilitres of 4 g/L NaOH-40 g/L Na_4_P_2_O_7_ mixed solution was added to the remained residue after the gold extraction of IECAS, and the subsequent operations were the same as methods I and II of Section 2.5.1, respectively.

#### 2.5.4. Iron-Manganese Oxide Bound State (IMOBS)

Fifty millilitres of 4 g/L 100 g/L ammonium citrate-40 g/L hydroxylamine hydrochloride mixed solution was added to the remained residue after the gold extraction of OMBS, and the subsequent operations were the same as methods I and II of Section 2.5.1, respectively.

#### 2.5.5. Naked or Seminaked State (NSNS)

Fifty millilitres of 5 g/L I_2_-15 g/L KI mixed solution was added to the remained residue after the gold extraction of IMOBS, and the subsequent operations were the same as methods I and II of Section 2.5.1, respectively.

#### 2.5.6. Carbonate Bound State (CBS)

Fifty millilitres of 4 mol/L HAc was added to the remained residue after the gold extraction of NSNS, and the subsequent operations were the same as methods I and II of Section 2.5.1, respectively.

#### 2.5.7. Sulfide Bound State (SBS)

Fifty millilitres of 5 mL/L Br_2_-100 g/L NaCl mixed solution was added to the remained residue after the gold extraction of CBS, and the subsequent operations were the same as methods I and II of Section 2.5.1, respectively.

#### 2.5.8. Insoluble Silicate State (ISS)

Methods I and II: 50 mL of 50% aqua regia and 10 mL HF were added to the remained residue after the gold extraction of SBS, and the subsequent operations were the same as the experimental procedure of total gold above, respectively.

### 2.6. Calibration Curves

Under the optimal conditions of the instrument in Table 1, two series of calibration curves were established separately with five concentrations of gold standard solutions and a blank. Curve I ranging from 0 to 10 *μ*g/mL for flame AAS was *y* = 0.0017 + 0.0522*x*, with a correlation coefficient of 0.9998, and curve II ranging from 0 to 50 ng/mL for graphite furnace AAS was *y* = 0.0003 + 0.0022*x*, with a correlation coefficient of 0.9996, which indicated that a good linear regression was established between the absorbances and the concentrations.

## 3. Results and Discussion

### 3.1. Validation of Analytical Methods

The accuracy and precision requirements of analytical methods can be validated by separately calculating the logarithmic deviation (Δ log *C*) and relative standard deviation (RSD) between the measured value and the standard of the CRM. The content of main constant and trace elements in CRMs GBW07105 and GBW07309 by XRF is listed in Table S1, the total gold content of CRMs GBW7247a and GBW07298a by AAS is shown in Table S2, and the relevant quality control parameters, Δ log *C* and RSD, could be evaluated from the following equations:

(1) $$\begin{array}{c} \Delta \;\mathrm{lg}\;C\;\;\left(\text{GBW}\right)=\left|\mathrm{lg}\;\overset{-}{C_{i}}-\mathrm{lg}\;C_{s}\right|,\\ \text{RSD }\left(\text{GBW}\right)=\frac{\sqrt{\sum_{i=1}^{n}{\left(C_{i}-C_{s}\right)}^{2}/\left(n-1\right)}}{C_{s}}\;\;\times 100\%, \end{array}$$

where *n* = 5 is the number of parallel experiments, *C*_*s*_ is the national standard value of the CRM, and *C*_*i*_ and $\bar{C_{i}}$ denote the single and mean measured value of the CRM, respectively.

In Tables S1 and S2, the Δ log *C* values were in the range of 0.16–2.52% and the RSDs were all less than 6.80%. The detection limits, estimated as three times the standard deviation of the blank, were 0.002 *μ*g/mL and 0.012 ng/mL for flame and graphite furnace AAS, respectively. According to the requirements of the geological and mineral industry standard DZ/T0011-2015 of the People's Republic of China, the detection limits of flame and graphite furnace AAS were lower than those required by the standard, and the Δlog*C* and RSD values of four CRMs were much smaller than the standard monitoring limits. These demonstrated that the accuracy and precision of the experimental method were high and satisfied the detection requirements.

### 3.2. Constant and Trace Element Content

Combined with the geological characteristics of the Laozuoshan gold deposit, the main constant elements Al, Ca, Fe, K, Mg, Na, and Si (expressed by oxide) and trace elements Co, Cu, Mn, Ni, Pb, Sn, and Zn of the sample were determined by XRF, and the results are shown in Tables S3 and 3. In the Laozuoshan gold mine, the main ore minerals were arsenopyrite, pyrrhotite, pyrite, and chalcopyrite, and the major gangue minerals were mainly quartz, diopside, and calcite; the measurement results were clearly related to these geological data. Among them, the rock ore sample LZY01 contained sulfide quartz vein, and its Fe, Cu, Pb, and Co contents were obviously high. LZY02 was granulite, its main mineral was quartz and feldspar, and its Si content was high. LZY03 had remarkable characteristics of brass mineralization, its Cu content was the highest among the six samples, and the content of other metal elements was also high. LZY04 with no obvious high content elements was a rock sample in the later stage of mineralization. LZY05 was an altered rock sample with a certain degree of quartzification during mineralization, and its Si content was high. The sediment sample LZS01 was collected from the upstream river sand near the original gold mining field, its Si content was high, and its Fe, Cu, Co, and Mn contents were low. Based on the above element content information, it was preliminarily predicted that the gold in the sample mainly existed in the SBS, the gold content of LZY01 and LZY03 would be higher than that of others, and the gold content in the sediment might be lower than that in the rock ore.

### 3.3. Total Gold Content

The measurement results of the total gold in the sample by AAS are shown in Tables S4 and 4. The total gold content from high to low was LZY01 > LZY03 > LZY02 > LZY04 > LZY05 > LZS01, which met the expectation of gold-related elements above and further indicated that gold was enriched in sulfide quartz veins. The gold in the sediment was derived from the debris formed by the weathering and erosion of the primary rock, but the tiny gold particles were carried away by water flow, so the gold content of the sediment was lower than that of the rock ore.

### 3.4. Comparison of Phase Analysis Results

**Figure 1:**
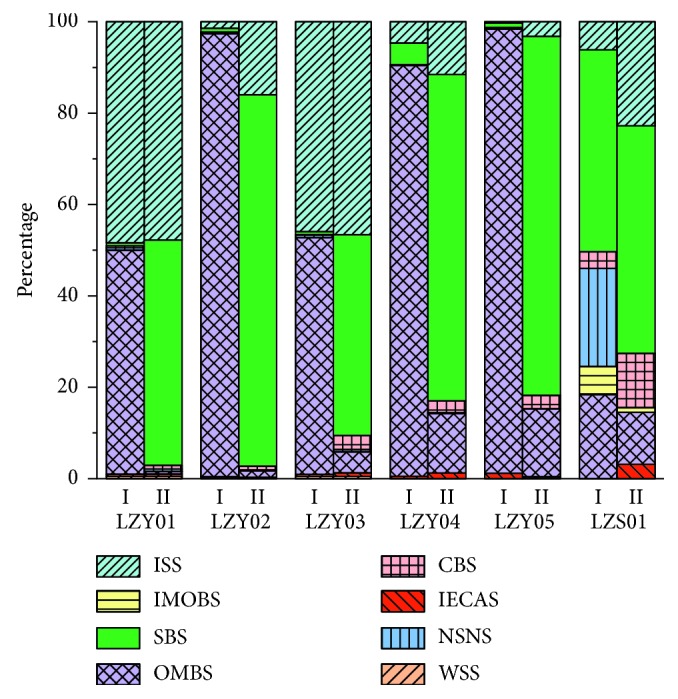
The histogram for the percentage of gold in each chemical phase (I: ultrasonic centrifugation; II: cyclotron oscillation; WSS: water-soluble state; IECAS: ion exchange and clay adsorption state; OMBS: organic matter bound state; IMOBS: iron-manganese oxide bound state; NSNS: naked or seminaked state; CBS: carbonate bound state; SBS: sulfide bound state; ISS: insoluble silicate state).

The gold content of each chemical phase in the sample was determined by AAS, the weighted value (the sum of eight phase gold contents), the percentage (the ratio of the phase gold content to the weighted value), and the leaching rate (the ratio of the weighted value to the total content) were calculated, and all the results are listed in Tables S5, S6, and 5. For more intuitive analysis and discussion of relevant data, a histogram for the percentage of gold in each chemical phase was plotted in Figure 1. Comparing the extraction effect of methods I and II for gold in each chemical phase, two common points were found. (1) The percentages of WSS, IECAS, IMOBS, and NSNS were low, and their maximum value did not exceed 2%, which indicated that these phase states contained less gold. (2) The gold percentage of ISS in LZY01 and LZY03 was close to 50% due to the inclusion of sulfide quartz veins. Except for above common points, there were significant differences between the two methods in Figure 1. For all the samples, the gold content of OMBS obtained by ultrasonic centrifugation was much higher than that by cyclotron oscillation, and the gold content of SBS extracted by cyclotron oscillation was much more than that by ultrasonic centrifugation. The average percentage of gold in OMBS extracted by ultrasonic centrifugation was up to 67.24%, and the average percentage of gold in SBS extracted by cyclotron oscillation was up to 62.37%. Moreover, the gold content of CBS extracted by cyclotron oscillation was also larger than that by ultrasonic centrifugation. Since the gold-bearing minerals in the Laozuoshan gold mining area were mainly arsenopyrite, pyrrhotite, pyrite, and chalcopyrite, and the content of sulfide in samples was high, thus, the gold extraction result of each chemical phase by cyclotron oscillation method was more in line with actual geological conditions. When extracting gold in OMBS, ultrasound would accelerate the internal motion and depolymerization of molecules, which might lead to the early release of the subsequent gold in CBS and SBS.

In addition, the gold content of SBS in sediment samples was the highest, indicating that the sediment system was affected by the primary halo, and its main phase composition was consistent with the mineral information in the mining area. Thus, the gold content and phase behavior of sediments could be used to preliminarily predict the rock and ore conditions in the corresponding mining areas, analyze and deduce the hard-to-obtain medium with the easily obtained sampling medium, estimate the deep mineral information with the aid of shallow source information, and then provide some theoretical guidance for the exploration of concealed gold deposits and the delineation of gold anomalies.

Comparing the gold leaching rates of the two methods in Table 5, the average leaching rate of ultrasonic centrifugation and cyclotron oscillation was 105.63% and 96.59%, respectively. The cyclotron oscillation method had a better leaching effect on gold, since its leaching rate was closer to 100%. The power and vibration frequency of method I were much higher than those of method II, but its extraction efficiency was not as good as method II. At the same time, the high power and high frequency vibration of method I also led to the enhanced cavitation, and the solution was easy to rise to high temperature, so the temperature needed to be strictly monitored and adjusted during the experiment process, which increased the complexity of the experiment, introduced more errors, and made the measurement results deviate from the geological conditions. While the experimental procedure of method II was simple and easy, the error was relatively reduced, so the cyclotron oscillation method had higher leaching ability and efficiency for gold, and its phase analysis result was more in line with the XRF data and the actual geological information. Furthermore, ultrasound would cause a certain degree of noise pollution, while the cyclotron oscillation method was greener and more environmentally friendly.

## 4. Conclusion

To reasonably analyze the chemical phase of gold in geological samples, good pretreatment and test methods are necessary. In this work, two pretreatment processes of ultrasonic centrifugation and cyclotron oscillation for the extraction of gold from eight chemical phases are compared, and the gold content is determined by AAS. It is found that the experiment steps are cumbersome, and it is easy to introduce more errors and cause a certain degree of noise pollution in ultrasonic centrifugation method. When extracting gold of OMBS, ultrasound accelerates the internal molecular movement and depolymerization and releases the subsequent gold of CBS and SBS in advance, which causes the high gold content of OMBS and the extraction results to be inconsistent with the actual situation. However, the main phase obtained by cyclotron oscillation method is SBS, which is consistent with the XRF analysis and the geological information of the Laozuoshan gold deposit. Moreover, the cyclotron oscillation has the advantages of simple experimental steps, relatively low error, and high gold leaching efficiency. Therefore, the cyclotron oscillation method is an accurate and quick pretreatment method, which meets the development requirements of green chemistry and environmental protection, and can be well suited for the determination of the chemical phase of gold in geological samples by AAS.

## Figures and Tables

**Table 1:** Instrument operating parameters.

Parameter	Setting
*XRF*	
Initialization element	Ag
Initialization channel	2210
Tube current	250 *μ*A
Tube voltage	40 kV
Counting rate	1
Vacuum time	25 s
Measure time	100 s
Replicates	3

*Flame AAS*	
Element	Au
Wavelength	242.8 nm
Spectral bandwidth	0.4 nm
Lamp current	2.0 mA
Filter coefficient	0.6
Integration time	3 s
Burner height	6 mm
Flame type	Air-C_2_H_2_

*Graphite furnace AAS*	
Element	Au
Wavelength	242.8 nm
Spectral bandwidth	0.4 nm
Lamp current	4.0 mA
Filter coefficient	0.1
Integration time	3 s
Injection volume	20 *μ*L
Measurement methods	Peak area

XRF: X-ray fluorescence spectrometry; AAS: atomic absorption spectrometry.

**Table 2:** Heating program for graphite furnace.

Step	Temperature (°C)	Heating time (s)	Holding time (s)	Internal gas flow (mL/min)
Dry	110	10	10	200
Ashing	600	10	15	200
Atomization	1800	0	3	0
Exclusion	1900	1	2	200

**Table 3:** The content of main constant and trace elements.

Constant element (unit: 10^−2^)							
Sample	Al_2_O_3_	CaO	Fe_2_O_3_	K_2_O	MgO	Na_2_O	SiO_2_
LZY01	3.140	7.598	34.367	0.188	0.586	2.213	44.198
LZY02	8.916	14.113	9.429	1.693	2.472	0.201	58.545
LZY03	7.945	20.719	8.677	0.166	1.746	0.668	47.335
LZY04	8.062	15.368	10.244	2.061	2.182	0.292	56.328
LZY05	12.504	9.485	3.513	1.600	1.136	1.968	61.755
LZS01	11.318	1.524	1.620	3.443	0.431	2.457	63.061

Trace element (unit: 10^−6^)							
Sample	Co	Cu	Mn	Ni	Pb	Sn	Zn

LZY01	255.089	900.836	1055.870	48.409	39.484	0.607	133.309
LZY02	19.689	120.047	2003.167	30.235	7.462	0.826	160.777
LZY03	18.392	1856.429	1848.804	11.438	14.410	0.742	134.596
LZY04	20.457	243.509	1973.857	20.027	4.505	0.784	143.916
LZY05	6.162	157.230	554.794	4.761	13.303	1.757	24.905
LZS01	1.475	26.164	117.641	2.562	48.004	0.052	297.242

**Table 4:** Analysis results of total gold.

Sample	LZY01	LZY02	LZY03	LZY04	LZY05	LZS01
Content (10^−6^)	46.985	3.980	26.060	1.840	1.490	0.176

**Table 5:** Analysis results of phase gold.

Sample	Method	WSS	IECAS	OMBS	IMOBS	NSNS	CBS	SBS	ISS	Weighted value	Leaching rate
LZY01	I	0.211	0.222	21.856	0.024	0.213	0.215	0.272	21.585	44.598	94.92
		(0.47)	(0.50)	(49.01)	(0.05)	(0.48)	(0.48)	(0.61)	(48.40)		
	II	0.224	0.238	0.240	0.026	0.216	0.384	22.261	21.572	45.161	96.12
		(0.50)	(0.53)	(0.53)	(0.06)	(0.48)	(0.85)	(49.29)	(47.77)		

LZY02	I	0.000	0.019	4.456	0.002	0.011	0.005	0.036	0.065	4.594	115.43
		(0.00)	(0.41)	(97.00)	(0.04)	(0.24)	(0.11)	(0.78)	(1.41)		
	II	0.006	0.006	0.051	0.007	0.003	0.032	3.079	0.605	3.789	95.20
		(0.16)	(0.16)	(1.35)	(0.18)	(0.08)	(0.84)	(81.26)	(15.97)		

LZY03	I	0.118	0.112	12.906	0.015	0.133	0.031	0.158	11.439	24.912	95.60
		(0.47)	(0.45)	(51.81)	(0.06)	(0.53)	(0.12)	(0.63)	(45.92)		
	II	0.125	0.194	1.163	0.014	0.115	0.792	11.153	11.817	25.373	97.36
		(0.49)	(0.76)	(4.58)	(0.06)	(0.45)	(3.12)	(43.96)	(46.57)		

LZY04	I	0.000	0.012	2.105	0.001	0.000	0.003	0.110	0.109	2.340	127.17
		(0.00)	(0.51)	(89.96)	(0.04)	(0.00)	(0.13)	(4.70)	(4.66)		
	II	0.000	0.019	0.203	0.003	0.000	0.041	1.113	0.180	1.559	84.73
		(0.00)	(1.22)	(13.02)	(0.19)	(0.00)	(2.63)	(71.39)	(11.55)		

LZY05	I	0.000	0.019	1.566	0.002	0.000	0.003	0.016	0.004	1.610	108.05
		(0.00)	(1.18)	(97.27)	(0.12)	(0.00)	(0.19)	(0.99)	(0.25)		
	II	0.000	0.006	0.213	0.001	0.000	0.042	1.129	0.046	1.437	96.44
		(0.00)	(0.42)	(14.82)	(0.07)	(0.00)	(2.92)	(78.57)	(3.20)		

LZS01	I	0.000	0.000	0.030	0.010	0.035	0.006	0.072	0.010	0.163	92.61
		(0.00)	(0.00)	(18.40)	(6.13)	(21.47)	(3.68)	(44.17)	(6.13)		
	II	0.000	0.006	0.022	0.002	0.000	0.023	0.096	0.044	0.193	109.66
		(0.00)	(3.11)	(11.40)	(1.04)	(0.00)	(11.92)	(49.74)	(22.80)		

I: ultrasonic centrifugation; II: cyclotron oscillation; WSS: water-soluble state; IECAS: ion exchange and clay adsorption state; OMBS: organic matter bound state; IMOBS: iron-manganese oxide bound state; NSNS: naked or seminaked state; CBS: carbonate bound state; SBS: sulfide bound state; ISS: insoluble silicate state. Among the eight phase states, the value outside the parentheses is the content of gold in each phase, and the unit is 10^−6^; the value in parentheses is the ratio of the phase gold content to the weighted value, and the unit is %. The weighted value is the sum of the eight phase gold contents, and the unit is 10^−6^; the leaching rate is the ratio of the weighted value to the total content, and the unit is %.

## Data Availability

The data used to support the findings of this study are included within the article.

## Supplementary Materials

Supplementary Materials Table S1: the measured values of main constant and trace elements for CRMs GBW07105 and GBW07309 by XRF. Table S2: the analysis results of total gold for CRMs GBW7247a and GBW07298a by AAS. Table S3: the measured values of main constant and trace elements for six samples by XRF. Table S4: the measured values of total gold for six samples by AAS. Table S5: the measured values of phase gold for six samples by the ultrasonic centrifugation method. Table S6: the measured values of phase gold for six samples by the cyclotron oscillation method.
