# Supplementary material for: Comparative Study on Two Pretreatment Processes for Chemical Phase Analysis of Gold in Geological Samples by Atomic Absorption Spectrometry
Source: J Anal Methods Chem. 2019 Jul 10;2019:1792792. doi: 10.1155/2019/1792792 (PMC6652044; doi:10.1155/2019/1792792)
Supplement: Supplementary Materials — Table S1: the measured values of main constant and trace elements for CRMs GBW07105 and GBW07309 by XRF. Table S2: the analysis results of total gold for CRMs GBW7247a and GBW07298a by AAS. Table S3: the measured values of main constant and trace elements for six samples by XRF. Table S4: the measured values of total gold for six samples by AAS. Table S5: the measured values of phase gold for six samples by the ultrasonic centrifugation method. Table S6: the measured values of phase gold for six samples by the cyclotron oscillation method. [file 1792792.f1.pdf]

Supplementary Materials

Comparative Study on Two Pretreatment Processes for Chemical Phase Analysis of Gold in Geological Samples by Atomic Absorption Spectrometry

Xiaodan Tang[1, 2], Hang Li[2], Hongyan Liu[1, 2], Bing Li[2], Yuyan Zhao[2], Jilong Lu[2], Jian Zhou[1, 3], Qingqing Liu[1, 3*]

[1]*Key Laboratory of Geochemical Exploration, Ministry of Land and Resources, Institute of Geophysical and Geochemical Exploration, Chinese Academy of Geological Sciences, Langfang 065000, China*

[2]*College of Geo-exploration Science and Technology, Jilin University, Changchun 130026, China*

[3]*UNESCO International Centre on Global-Scale Geochemistry, Langfang 065000, China*

Correspondence should be addressed to Qingqing Liu. E-mail: liuqingqing@igge.cn

TABLE S1: The measured values of main constant and trace elements for CRMs GBW07105 and GBW07309 by XRF

CRM	Element	C_1	C_2	C_3	C_4	C_5	$\overline{C_i}$	C_s	ΔlogC (GBW)/%	RSD (GBW)/%
GBW07105	Al_2O_3	13.750	13.788	13.825	13.734	13.791	13.778	13.830	0.16	0.50
	CaO	9.072	9.068	9.070	9.074	9.069	9.071	8.810	1.27	3.31
	Fe_2O_3	14.022	13.996	13.973	13.982	13.970	13.989	13.400	1.87	4.91
	K_2O	2.402	2.395	2.395	2.396	2.393	2.396	2.320	1.40	3.67
	MgO	7.701	7.708	7.675	7.688	7.684	7.691	7.770	0.44	1.15
	Na_2O	3.272	3.246	3.258	3.205	3.262	3.248	3.380	1.72	4.42
	SiO_2	44.289	44.317	44.292	44.301	44.251	44.290	44.640	0.34	0.88
	Co	47.851	47.363	47.977	47.186	47.997	47.675	46.500	1.08	2.94
	Cu	50.429	50.819	50.227	50.191	50.699	50.473	49.000	1.29	3.41
	Mn	1264.066	1262.227	1246.589	1266.555	1248.527	1257.593	1310.000	1.77	4.53
	Ni	149.885	148.628	148.574	148.058	146.607	148.350	140.000	2.52	6.72
	Pb	7.229	7.073	7.356	7.834	7.039	7.306	7.000	1.86	6.71
	Sn	2.164	2.092	2.056	2.123	2.095	2.106	2.000	2.24	6.26
	Zn	157.781	159.366	157.984	156.587	159.171	158.178	150.000	2.31	6.14
GBW07309	Al_2O_3	10.117	10.836	10.727	10.897	10.673	10.650	10.580	0.29	3.03
	CaO	5.068	5.097	5.139	5.036	5.129	5.094	5.350	2.13	5.42
	Fe_2O_3	5.118	4.754	4.371	4.510	4.924	4.735	4.860	1.13	6.85
	K_2O	1.923	1.985	2.009	2.155	1.948	2.004	1.990	0.31	4.62
	MgO	2.336	2.133	2.434	2.400	2.213	2.303	2.390	1.61	6.69
	Na_2O	1.312	1.448	1.342	1.454	1.340	1.379	1.440	1.88	6.62
	SiO_2	63.363	64.318	63.363	60.024	61.932	62.600	64.890	1.56	4.71
	Co	13.182	13.446	13.895	14.278	13.710	13.702	14.400	2.16	6.15
	Cu	30.473	30.477	30.455	30.445	30.438	30.457	32.000	2.15	5.39
	Mn	624.537	625.506	626.018	622.740	623.317	624.424	620.000	0.31	0.83
	Ni	34.100	33.958	33.505	33.483	34.173	33.844	32.000	2.43	6.52
	Pb	23.737	24.124	24.186	23.909	23.773	23.946	23.000	1.75	4.68
	Sn	2.392	2.566	2.342	2.528	2.633	2.492	2.600	1.84	6.58
	Zn	72.830	76.583	73.221	78.899	71.376	74.582	78.000	1.95	6.29

CRM: certified reference material; Δlog$C$ (GBW): the logarithmic deviation of the CRM; RSD (GBW): the relative standard deviation of the CRM; C_s: the national standard value of the CRM; $\overline{C_i}$: the mean measured value of the CRM. The units of constant elements (Al_2O_3, CaO, Fe_2O_3, K_2O, MgO, Na_2O and SiO_2) and trace elements (Co, Cu, Mn, Ni, Pb, Sn and Zn) are 10^{-2} and 10^{-6}, respectively.

TABLE S2: The measured values of total gold for CRMs GBW7247a and GBW07298a by AAS

CRM	GBW07298a	GBW7247a
$C_1/10^{-6}$	30.165	0.050
$C_2/10^{-6}$	29.470	0.046
$C_3/10^{-6}$	32.205	0.049
$C_4/10^{-6}$	31.430	0.050
$C_5/10^{-6}$	30.905	0.047
$\overline{C_i}/10^{-6}$	30.835	0.048
$C_s/10^{-6}$	31.900	0.050
ΔlogC (GBW)/%	1.47	1.41
RSD (GBW)/%	5.01	5.10

CRM: certified reference material; Δlog$C$ (GBW): the logarithmic deviation of the CRM; RSD (GBW): the relative standard deviation of the CRM; C_s: the national standard value of the CRM; $\overline{C_i}$: the mean measured value of the CRM.

TABLE S3: The measured values of main constant and trace elements for six samples by XRF

Sample	Element	Measured value 1	Measured value 2	Measured value 3	Measured value 4	Measured value 5	Average value	SD	RSD/%
LZY01	Al_2O_3	3.265	3.205	3.002	3.144	3.087	3.140	0.102	3.25
	CaO	7.787	7.049	7.637	7.803	7.715	7.598	0.314	4.13
	Fe_2O_3	34.812	34.083	34.610	33.317	35.016	34.367	0.682	1.98
	K_2O	0.199	0.194	0.185	0.175	0.188	0.188	0.009	5.01
	MgO	0.582	0.605	0.607	0.528	0.605	0.586	0.034	5.76
	Na_2O	2.230	2.118	2.198	2.135	2.385	2.213	0.106	4.81
	SiO_2	44.257	44.176	44.327	44.117	44.114	44.198	0.092	0.21
	Co	257.154	258.306	255.469	253.664	250.851	255.089	2.947	1.16
	Cu	944.381	860.877	897.531	890.029	911.360	900.836	30.542	3.39
	Mn	1055.752	1055.908	1055.667	1056.040	1055.982	1055.870	0.156	0.01
	Ni	47.272	50.741	50.819	43.832	49.381	48.409	2.934	6.06
	Pb	36.964	36.522	42.080	41.053	40.799	39.484	2.552	6.46
	Sn	0.604	0.608	0.609	0.610	0.604	0.607	0.003	0.45
	Zn	133.598	133.021	133.274	133.529	133.121	133.309	0.251	0.19
LZY02	Al_2O_3	8.096	8.857	9.037	9.132	9.457	8.916	0.507	5.69
	CaO	14.122	13.971	13.922	14.695	13.858	14.113	0.339	2.40
	Fe_2O_3	9.386	9.466	8.940	9.768	9.587	9.429	0.309	3.28
	K_2O	1.687	1.785	1.648	1.720	1.626	1.693	0.063	3.71
	MgO	2.576	2.587	2.381	2.254	2.564	2.472	0.149	6.01
	Na_2O	0.202	0.188	0.199	0.206	0.212	0.201	0.009	4.43
	SiO_2	55.474	57.737	60.724	56.634	62.154	58.545	2.807	4.79
	Co	20.048	18.850	21.041	18.248	20.260	19.689	1.125	5.71
	Cu	122.491	113.754	115.615	121.316	127.056	120.047	5.384	4.48
	Mn	2129.355	1976.352	2152.936	1895.989	1861.202	2003.167	132.961	6.64
	Ni	30.467	31.619	28.628	30.054	30.406	30.235	1.074	3.55
	Pb	7.603	7.161	7.174	7.539	7.832	7.462	0.290	3.89
	Sn	0.823	0.872	0.784	0.867	0.782	0.826	0.044	5.27
	Zn	154.075	152.898	155.458	164.848	176.609	160.777	10.031	6.24
LZY03	Al_2O_3	8.015	7.962	7.757	7.893	8.100	7.945	0.130	1.63
	CaO	20.370	20.381	20.496	20.768	21.578	20.719	0.507	2.45
	Fe_2O_3	8.447	8.674	8.503	8.718	9.043	8.677	0.234	2.69
	K_2O	0.167	0.165	0.164	0.169	0.167	0.166	0.002	1.26
	MgO	1.751	1.790	1.723	1.694	1.771	1.746	0.038	2.18
	Na_2O	0.678	0.664	0.675	0.652	0.674	0.668	0.010	1.57
	SiO_2	46.595	45.643	49.160	46.851	48.426	47.335	1.429	3.02
	Co	17.832	18.078	19.581	18.000	18.467	18.392	0.705	3.83
	Cu	1980.223	1860.646	1851.294	1792.080	1797.899	1856.429	75.723	4.08
	Mn	1817.844	1847.943	1921.467	1790.239	1866.528	1848.804	49.958	2.70
	Ni	12.300	10.484	10.717	12.127	11.565	11.438	0.816	7.13
	Pb	14.498	14.563	13.633	15.508	13.848	14.410	0.734	5.10
	Sn	0.759	0.700	0.785	0.733	0.730	0.742	0.032	4.34
	Zn	136.609	125.458	138.306	133.043	139.564	134.596	5.666	4.21
LZY04	Al_2O_3	7.927	8.135	8.149	8.092	8.009	8.062	0.093	1.16
	CaO	15.407	15.249	15.299	15.537	15.351	15.368	0.111	0.72
	Fe_2O_3	10.258	10.341	10.236	10.185	10.199	10.244	0.062	0.60
	K_2O	2.023	2.020	2.072	2.098	2.094	2.061	0.038	1.84
	MgO	2.104	2.259	2.264	2.177	2.106	2.182	0.078	3.58
	Na_2O	0.296	0.283	0.279	0.295	0.307	0.292	0.011	3.85
	SiO_2	56.380	56.195	56.342	56.397	56.325	56.328	0.080	0.14
	Co	19.829	20.867	20.974	20.755	19.858	20.457	0.565	2.76
	Cu	230.112	259.772	245.536	231.434	250.689	243.509	12.703	5.22
	Mn	1969.375	1975.407	1943.403	1941.972	2039.126	1973.857	39.455	2.00
	Ni	20.310	18.815	20.220	21.109	19.682	20.027	0.848	4.23
	Pb	4.660	4.111	4.508	4.708	4.536	4.505	0.235	5.22

	Sn	0.803	0.748	0.746	0.830	0.793	0.784	0.036	4.65
	Zn	144.568	139.569	139.831	146.275	149.336	143.916	4.212	2.93
LZY05	Al_2O_3	12.284	12.537	12.373	12.481	12.843	12.504	0.213	1.71
	CaO	9.333	9.475	9.525	9.655	9.437	9.485	0.119	1.25
	Fe_2O_3	3.636	3.520	3.555	3.482	3.372	3.513	0.097	2.77
	K_2O	1.603	1.600	1.602	1.595	1.600	1.600	0.003	0.20
	MgO	1.073	1.187	1.171	1.162	1.085	1.136	0.052	4.62
	Na_2O	1.924	1.969	1.940	2.057	1.950	1.968	0.053	2.68
	SiO_2	61.897	61.905	60.988	61.971	62.014	61.755	0.432	0.70
	Co	5.968	6.464	6.303	6.042	6.032	6.162	0.212	3.44
	Cu	160.476	150.965	156.675	157.624	160.410	157.230	3.884	2.47
	Mn	540.555	545.632	569.388	589.756	528.636	554.794	24.527	4.42
	Ni	4.540	4.707	4.708	4.774	5.076	4.761	0.196	4.12
	Pb	13.510	12.984	13.375	13.664	12.984	13.303	0.309	2.32
	Sn	1.675	1.864	1.819	1.739	1.688	1.757	0.082	4.68
	Zn	24.902	26.200	24.172	23.820	25.432	24.905	0.958	3.85
LZS01	Al_2O_3	11.065	11.432	11.076	11.455	11.563	11.318	0.231	2.04
	CaO	1.536	1.518	1.525	1.529	1.511	1.524	0.010	0.64
	Fe_2O_3	1.628	1.610	1.612	1.632	1.620	1.620	0.009	0.57
	K_2O	3.483	3.429	3.396	3.470	3.438	3.443	0.034	1.00
	MgO	0.431	0.433	0.435	0.429	0.427	0.431	0.003	0.78
	Na_2O	2.424	2.450	2.485	2.474	2.453	2.457	0.024	0.96
	SiO_2	63.201	63.191	62.903	62.920	63.092	63.061	0.144	0.23
	Co	1.438	1.483	1.475	1.460	1.518	1.475	0.030	2.01
	Cu	27.050	27.209	24.938	26.590	25.032	26.164	1.100	4.20
	Mn	117.755	121.233	110.754	117.385	121.075	117.641	4.249	3.61
	Ni	2.476	2.710	2.658	2.537	2.428	2.562	0.119	4.66
	Pb	46.642	48.792	48.543	49.034	47.010	48.004	1.097	2.29
	Sn	0.050	0.056	0.050	0.052	0.055	0.052	0.003	4.92
	Zn	313.682	296.468	290.473	282.647	302.938	297.242	11.855	3.99

The units of measured, average and SD values for constant elements (Al_2O_3, CaO, Fe_2O_3, K_2O, MgO, Na_2O and SiO_2) and trace elements (Co, Cu, Mn, Ni, Pb, Sn and Zn) are 10^{-2} and 10^{-6}, respectively.

TABLE S4: The measured values of total gold for six samples by AAS

Sample	LZY01	LZY02	LZY03	LZY04	LZY05	LZS01
Measured value 1/10^{-6}	47.385	4.066	25.690	1.945	1.398	0.169
Measured value 2/10^{-6}	46.531	3.894	26.050	1.732	1.562	0.191
Measured value 3/10^{-6}	45.923	3.988	25.779	1.921	1.493	0.178
Measured value 4/10^{-6}	47.028	3.775	25.755	1.870	1.562	0.169
Measured value 5/10^{-6}	48.060	4.179	27.027	1.732	1.434	0.173
Average value/10^{-6}	46.985	3.980	26.060	1.840	1.490	0.176
SD/10^{-6}	0.814	0.155	0.558	0.102	0.074	0.009
RSD/%	1.73	3.90	2.14	5.56	4.98	5.21

TABLE S5: The measured values of phase gold for six samples by ultrasonic centrifugation method

Sample	Phase	Measured value 1/10^{-6}	Measured value 2/10^{-6}	Measured value 3/10^{-6}	Measured value 4/10^{-6}	Measured value 5/10^{-6}	Average value/10^{-6}	SD/10^{-6}	RSD/%
LZY01	WSS	0.210	0.201	0.233	0.210	0.201	0.211	0.013	6.21
	IECAS	0.236	0.220	0.204	0.215	0.236	0.222	0.014	6.24
	OMBS	22.133	21.548	21.416	21.689	22.494	21.856	0.447	2.05
	IMOBS	0.023	0.026	0.023	0.026	0.023	0.024	0.002	6.79
	NSNS	0.201	0.233	0.215	0.201	0.215	0.213	0.013	6.19
	CBS	0.210	0.215	0.210	0.220	0.220	0.215	0.005	2.33
	SBS	0.288	0.271	0.256	0.256	0.288	0.272	0.016	5.89
	ISS	19.984	23.726	22.243	21.416	20.558	21.585	1.472	6.82
LZY02	WSS	0.000	0.000	0.000	0.000	0.000	0.000	0.000	0.00
	IECAS	0.019	0.019	0.019	0.019	0.017	0.019	0.001	4.81
	OMBS	4.523	4.261	4.335	4.638	4.523	4.456	0.154	3.45
	IMOBS	0.002	0.002	0.002	0.002	0.002	0.002	0.000	0.00
	NSNS	0.011	0.010	0.011	0.011	0.010	0.011	0.001	5.17
	CBS	0.005	0.005	0.005	0.005	0.005	0.005	0.000	0.00
	SBS	0.034	0.038	0.038	0.034	0.034	0.036	0.002	6.15
	ISS	0.062	0.066	0.064	0.063	0.068	0.065	0.002	3.73
LZY03	WSS	0.114	0.122	0.129	0.111	0.114	0.118	0.007	6.26
	IECAS	0.111	0.108	0.114	0.111	0.111	0.112	0.002	1.34
	OMBS	12.189	13.306	11.839	14.027	13.169	12.906	0.886	6.86
	IMOBS	0.014	0.016	0.014	0.016	0.015	0.015	0.001	6.67
	NSNS	0.124	0.141	0.134	0.124	0.141	0.133	0.009	6.42
	CBS	0.029	0.033	0.032	0.030	0.032	0.031	0.002	5.27
	SBS	0.159	0.167	0.153	0.153	0.159	0.158	0.006	3.64
	ISS	11.266	12.027	11.481	11.114	11.308	11.439	0.354	3.09
LZY04	WSS	0.000	0.000	0.000	0.000	0.000	0.000	0.000	0.00
	IECAS	0.011	0.012	0.012	0.012	0.011	0.012	0.001	4.72
	OMBS	2.176	2.161	1.950	2.043	2.193	2.105	0.105	4.97
	IMOBS	0.001	0.001	0.001	0.001	0.001	0.001	0.000	0.00
	NSNS	0.000	0.000	0.000	0.000	0.000	0.000	0.000	0.00
	CBS	0.003	0.003	0.003	0.003	0.003	0.003	0.000	0.00
	SBS	0.108	0.111	0.111	0.108	0.112	0.110	0.002	1.70
	ISS	0.114	0.108	0.106	0.108	0.111	0.109	0.003	2.86
LZY05	WSS	0.000	0.000	0.000	0.000	0.000	0.000	0.000	0.00
	IECAS	0.019	0.017	0.019	0.020	0.019	0.019	0.001	5.83
	OMBS	1.723	1.562	1.616	1.493	1.434	1.566	0.112	7.14
	IMOBS	0.002	0.002	0.002	0.002	0.002	0.002	0.000	0.00
	NSNS	0.000	0.000	0.000	0.000	0.000	0.000	0.000	0.00
	CBS	0.003	0.003	0.003	0.003	0.003	0.003	0.000	0.00
	SBS	0.017	0.015	0.017	0.017	0.015	0.016	0.001	6.76
	ISS	0.004	0.004	0.004	0.004	0.004	0.004	0.000	0.00
LZS01	WSS	0.000	0.000	0.000	0.000	0.000	0.000	0.000	0.00
	IECAS	0.000	0.000	0.000	0.000	0.000	0.000	0.000	0.00
	OMBS	0.028	0.031	0.030	0.029	0.031	0.030	0.001	4.38
	IMOBS	0.010	0.011	0.010	0.011	0.010	0.010	0.001	5.27
	NSNS	0.033	0.037	0.034	0.036	0.036	0.035	0.002	4.67
	CBS	0.006	0.006	0.006	0.006	0.006	0.006	0.000	0.00
	SBS	0.065	0.077	0.074	0.075	0.071	0.072	0.005	6.45
	ISS	0.009	0.010	0.009	0.010	0.010	0.010	0.001	5.71

WSS: water soluble state; IECAS: ion exchange and clay adsorption state; OMBS: organic matter bound state; IMOBS: iron-manganese oxide bound state; NSNS: naked or semi-naked state; CBS: carbonate bound state; SBS: sulfide bound state; ISS: insoluble silicate state.

TABLE S6: The measured values of phase gold for six samples by cyclotron oscillation method

Sample	Phase	Measured value 1/10^{-6}	Measured value 2/10^{-6}	Measured value 3/10^{-6}	Measured value 4/10^{-6}	Measured value 5/10^{-6}	Average value/10^{-6}	SD/10^{-6}	RSD/%
LZY01	WSS	0.233	0.210	0.210	0.233	0.236	0.224	0.013	5.88
	IECAS	0.243	0.220	0.243	0.243	0.243	0.238	0.010	4.31
	OMBS	0.243	0.236	0.236	0.243	0.243	0.240	0.004	1.69
	IMOBS	0.025	0.028	0.026	0.025	0.028	0.026	0.002	5.74
	NSNS	0.220	0.201	0.233	0.204	0.220	0.216	0.013	6.09
	CBS	0.401	0.386	0.366	0.401	0.366	0.384	0.018	4.57
	SBS	20.558	22.494	22.133	21.584	24.537	22.261	1.467	6.59
	ISS	22.494	22.243	21.416	20.017	21.689	21.572	0.969	4.49
LZY02	WSS	0.006	0.006	0.006	0.006	0.006	0.006	0.000	0.00
	IECAS	0.006	0.006	0.006	0.006	0.006	0.006	0.000	0.00
	OMBS	0.054	0.049	0.049	0.054	0.049	0.051	0.003	5.37
	IMOBS	0.007	0.006	0.007	0.007	0.007	0.007	0.000	6.58
	NSNS	0.003	0.003	0.003	0.003	0.003	0.003	0.000	0.00
	CBS	0.030	0.035	0.030	0.033	0.033	0.032	0.002	6.73
	SBS	3.103	3.217	3.038	2.879	3.159	3.079	0.130	4.23
	ISS	0.603	0.585	0.591	0.659	0.585	0.605	0.031	5.17
LZY03	WSS	0.122	0.129	0.129	0.122	0.122	0.125	0.004	3.07
	IECAS	0.201	0.190	0.201	0.204	0.175	0.194	0.012	6.17
	OMBS	1.229	1.133	1.195	1.124	1.133	1.163	0.047	4.01
	IMOBS	0.016	0.014	0.014	0.014	0.014	0.014	0.001	6.21
	NSNS	0.111	0.122	0.122	0.111	0.111	0.115	0.006	5.22
	CBS	0.817	0.726	0.770	0.826	0.820	0.792	0.043	5.43
	SBS	11.114	10.557	11.308	11.266	11.481	11.153	0.408	3.66
	ISS	12.027	11.481	12.431	11.308	11.839	11.817	0.445	3.77
LZY04	WSS	0.000	0.000	0.000	0.000	0.000	0.000	0.000	0.00
	IECAS	0.020	0.019	0.017	0.020	0.019	0.019	0.001	6.45
	OMBS	0.201	0.210	0.204	0.201	0.201	0.203	0.004	1.92
	IMOBS	0.003	0.003	0.003	0.003	0.003	0.003	0.000	0.00
	NSNS	0.000	0.000	0.000	0.000	0.000	0.000	0.000	0.00
	CBS	0.039	0.043	0.040	0.039	0.043	0.041	0.002	5.02
	SBS	1.133	1.043	1.220	1.043	1.124	1.113	0.074	6.63
	ISS	0.191	0.191	0.178	0.170	0.170	0.180	0.011	5.87
LZY05	WSS	0.000	0.000	0.000	0.000	0.000	0.000	0.000	0.00
	IECAS	0.006	0.006	0.006	0.006	0.006	0.006	0.000	0.00
	OMBS	0.204	0.233	0.210	0.201	0.215	0.213	0.013	5.94
	IMOBS	0.001	0.001	0.001	0.001	0.001	0.001	0.000	0.00
	NSNS	0.000	0.000	0.000	0.000	0.000	0.000	0.000	0.00
	CBS	0.039	0.044	0.043	0.040	0.043	0.042	0.002	5.19
	SBS	1.043	1.124	1.223	1.034	1.220	1.129	0.092	8.11
	ISS	0.044	0.050	0.046	0.048	0.044	0.046	0.003	5.62
LZS01	WSS	0.000	0.000	0.000	0.000	0.000	0.000	0.000	0.00
	IECAS	0.006	0.006	0.006	0.006	0.006	0.006	0.000	0.00
	OMBS	0.023	0.022	0.024	0.021	0.022	0.022	0.001	5.09
	IMOBS	0.002	0.002	0.002	0.002	0.002	0.002	0.000	0.00
	NSNS	0.000	0.000	0.000	0.000	0.000	0.000	0.000	0.00
	CBS	0.024	0.021	0.023	0.022	0.024	0.023	0.001	5.72
	SBS	0.090	0.102	0.097	0.092	0.097	0.096	0.005	4.94
	ISS	0.040	0.047	0.045	0.045	0.043	0.044	0.003	6.01

WSS: water soluble state; IECAS: ion exchange and clay adsorption state; OMBS: organic matter bound state; IMOBS: iron-manganese oxide bound state; NSNS: naked or semi-naked state; CBS: carbonate bound state; SBS: sulfide bound state; ISS: insoluble silicate state.
